# Low-cost modular system for magneto-rheological testing in rheometers using 3D printing

**DOI:** 10.1016/j.ohx.2026.e00853

**Published:** 2026-10-23

**Authors:** Laura Álvarez-Gil, Juan José Alcalde-Castro, Wilmer Saldarriaga-Agudelo, Néstor Rojas-Reyes

**Affiliations:** a Departamento de Materiales y Minerales, Facultad de Minas, Universidad Nacional de Colombia, Medellín, Colombia; b Departamento de Ingeniería Mecánica, Facultad de Ingeniería, Institución Universitaria Pascual Bravo, Medellín, Colombia; c Departamento de Ingeniería Mecánica, Facultad de Minas, Universidad Nacional de Colombia, Medellín, Colombia; d Departamento de Física, Facultad de Ciencias, Universidad Nacional de Colombia, Medellín, Colombia

**Keywords:** Magnetorheology, Ferrofluids, 3D Printing, Low-cost system, Rheological characterization

## Abstract

In this study, a low-cost modular system was developed for the rheological characterization of ferrofluids under applied magnetic fields. The 3D-printed system is compatible with conventional rotational rheometers and includes two complementary components: a modular magnetic-field application accessory based on neodymium magnets and a gauge element for vectorial characterization of the magnetic field in a Cartesian coordinate system. This configuration enables alignment between the applied field and the rheometric geometry and provides clearance for future integration of thermal insulation or heating elements. Experimental validation was performed using magnetite ferrofluids in an ethylene glycol base at six solid concentrations ranging from 0.01 to 1 % Cv. The rheological response was evaluated under different magnetic field intensities, and measurements were performed in triplicate to assess repeatability and experimental dispersion. The results show that the proposed system is a practical and accessible platform for evaluating field-induced rheological variations in magnetic nanofluids within the tested operating range.

## Graphical abstract

**Figure:**
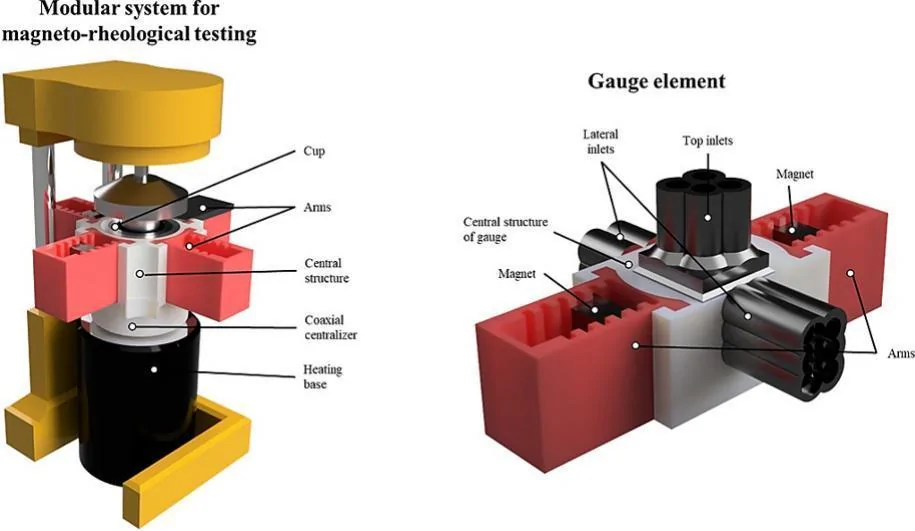

## 1 Hardware in context

*Ferrofluids are colloidal suspensions of magnetic nanoparticles in a liquid medium; these have recently emerged as highly versatile materials with applications across various fields* [1]*. Their distinctive ability to respond to magnetic fields and their fluid properties endow them with various functionalities. Ferrofluids are used as contrast agents in the medical field to enhance magnetic resonance imaging. Their use in targeted drug delivery systems is also being investigated* [2]*. They are used in engineering for developing magnetic seals, vibration dampers, and smart lubricants with tunable viscosity* [3]*. Ferrofluids also have the potential to revolutionize solar energy systems* [4] *and considerably enhance heat transfer efficiency in thermal engineering applications such as cooling systems for electronics* [5]*. They can be applied in material separation, sensor technology, and energy harvesting devices* [6]*. Ferrofluids can tune their viscosity and thermal conductivity when exposed to magnetic fields, making them useful in a wide range of engineering and technological applications* [7]*.*

*In this work, the term ferrofluid refers to a stable colloidal suspension of magnetic nanoparticles dispersed in a carrier liquid. Although the particles used in the validation tests, with sizes in the range of 60–100 nm, are larger than those commonly reported for classical ferrofluids, they remain within the nanoscale and are considerably smaller than the micrometer-sized particles typically used in conventional magnetorheological fluids. Therefore, the proposed system is primarily intended for the rheological characterization of magnetic nanofluids or ferrofluids under externally applied magnetic fields, while it may also be adapted to other magnetic suspensions depending on particle size, suspension stability, rheometer geometry, and operating conditions*. *The main specifications of the developed hardware are summarized in* Table 1. *The design files of the developed hardware are summarized in* Table 2. *The components, quantities, materials, and associated costs of the developed hardware are summarized in* Table 3.

**Table 1:** Specifications of the developed hardware.

Hardware name	*Modular system for the magnetorheological characterization of ferrofluids*
Subject area	• Engineering and materials science
Hardware type	• Mechanical engineering and materials science
Closest commercial analog	*No commercial analog is available.*
Open source license	*CC BY 4.0*
Cost of hardware	*USD 161*
Source file repository	*https://doi.org/10.17632/5tpzgkvj3w.2*

**Table 2:** Summary of design files.

**Design file name**	**File type**	**Open source license**	**Location of the file**
Central structure	STL	*CC BY 4.0*	[8]
Coaxial centralizer	STL	*CC BY 4.0*	[8]
Arms	STL	*CC BY 4.0*	[8]
Capsules	STL	*CC BY 4.0*	[8]
Capsule covers	STL	*CC BY 4.0*	[8]
Arm covers	STL	*CC BY 4.0*	[8]
Central structure of gauge	STL	*CC BY 4.0*	[8]
Axial inlet	STL	*CC BY 4.0*	[8]
Peripheral inlet	STL	*CC BY 4.0*	[8]
Top inlet 1	STL	*CC BY 4.0*	[8]
Top inlet 2	STL	*CC BY 4.0*	[8]
Top inlet 3	STL	*CC BY 4.0*	[8]

**Table 3:** Bill of materials.

**Designator**	**Component**	**Number**	**Cost per unit (USD)**	**Total cost (USD)**	**Source of materials**	**Material type**
Central structure	Central structure	1	4.14	4.14	Creality	Polymer
Coaxial centralizer	Coaxial centralizer	1	3.55	3.55	Creality	Polymer
Arms	Arm	4	2.30	9.20	Creality	Polymer
Capsules	Capsule	4	0.25	1.00	Creality	Polymer
Capsule covers	Capsule cover	4	0.12	0.46	Creality	Polymer
Arm covers	Arm cover	4	0.37	1.47	Creality	Polymer
Central structure of the gauge	Central structure of the gauge	1	5.91	5.91	Creality	Polymer
Axial inlet	Axial inlet	1	0.78	0.78	Creality	Polymer
Peripheral inlet	Peripheral inlet	1	1.04	1.04	Creality	Polymer
Top inlet 1	Top inlet 1	1	1.66	1.66	Creality	Polymer
Top inlet 2	Top inlet 2	1	1.38	1.38	Creality	Polymer
Top inlet 3	Top inlet 3	1	1.13	1.13	Creality	Polymer
Gaussmeter	Gaussmeter	1	89.00	89.00	Gain Express	Other
Magnets	Magnets	4	10.00	40.00	Nemco de Colombia	Metal

*Rheological characterization—a critical aspect in the study of ferrofluids—depends on many variables such as particle size distribution, suspended solid concentration, interaction with surfactants, surface free energy, temperature, and impurities in the crystalline structure* [9]*. The intensity and direction of the applied magnetic field directly influence the response of ferrofluids and impact their viscosity, stability, and heat-transfer capacity* [10]*. Due to the complexity of these factors, no universal mathematical relationships that accurately describe their flow behavior have been proposed. Therefore, specific rheological models must be developed to predict their behavior under different operating conditions* [9]*.*

*Flow properties of materials are characterized using rotational rheometers. These instruments use various sample dosages and correlate rotational speed with shear rate and applied torque with shear stress using geometric constants. Based on these correlations, stress versus shear rate curves can be developed to determine the viscosity at specific shear rates or temperatures* [11]*.*

*The rheological properties of ferrofluids under the influence of magnetic fields can be determined using specialized commercial equipment, e.g., the Anton Paar magnetorheological characterization accessory that is compatible with MCR rheometers* [12]*. However, it has significant limitations due to its high cost and poor compatibility with other commercial rheometer references, which limits its accessibility to laboratories with different measurement systems. These limitations can be addressed by developing an accessory that facilitates the characterization of ferrofluids via versatile and accessible experimental setups.*

## 2 Hardware description

*Herein, a modular accessory was developed to apply magnetic fields during rheological tests of ferrofluids. The accessory consists of a central structure coupled to a positioning base and four arms for locating permanent magnets. Each arm includes six guides for magnet capsules, allowing different magnet-to-sample distances and, consequently, different magnetic field intensities.*

*The central structure includes a compartment designed to accommodate a fluid-containing cup with an outer diameter of up to 83 mm. This geometry leaves space between the cup and the magnetic assembly for a non-magnetic insulating layer or future thermal-control elements.*

*The printed accessory does not contact the tested fluid; the sample remains confined within the rheometer cup, while the accessory acts as an external magnetic-field positioning system. Thus, compatibility with different magnetic suspensions depends mainly on the rheometer geometry, cup dimensions, magnetic field configuration, suspension stability, and operating conditions. The system may therefore be adapted to magnetic nanofluids or other magnetic suspensions, provided that sedimentation, wall slip, or excessive structuring do not compromise the rheological measurement.*

*For the Rheotest RN 5.1 configuration, the cup outer diameter is 43 mm; therefore, the 83-mm compartment provides an available radial clearance of approximately 20 mm. This space can accommodate a non-magnetic insulating layer, such as silicone or silicone-foam sheets, and may also allow future integration of a thin heating jacket with insulation. Because heating reduces the effective insulation thickness, elevated-temperature operation should be performed only with temperature monitoring of the cup, magnets, and PLA components, keeping the printed structure below a conservative limit of approximately 45 °C and the magnets within their safe operating range.*

*The system incorporates a centralizing element that functions as a coaxial alignment platform. This element ensures that the rotation axis of the rotor is aligned with the symmetry axis of the accessory, and an adequate insertion depth of the measuring cup is maintained inside the generated magnetic field.* Fig. 1*(a) shows the assembled system along with the modular setup used for magnetorheological characterization, while* Fig. 1*(b) provides a cross-sectional view identifying the main components of the assembly.*

**Fig. 1:**
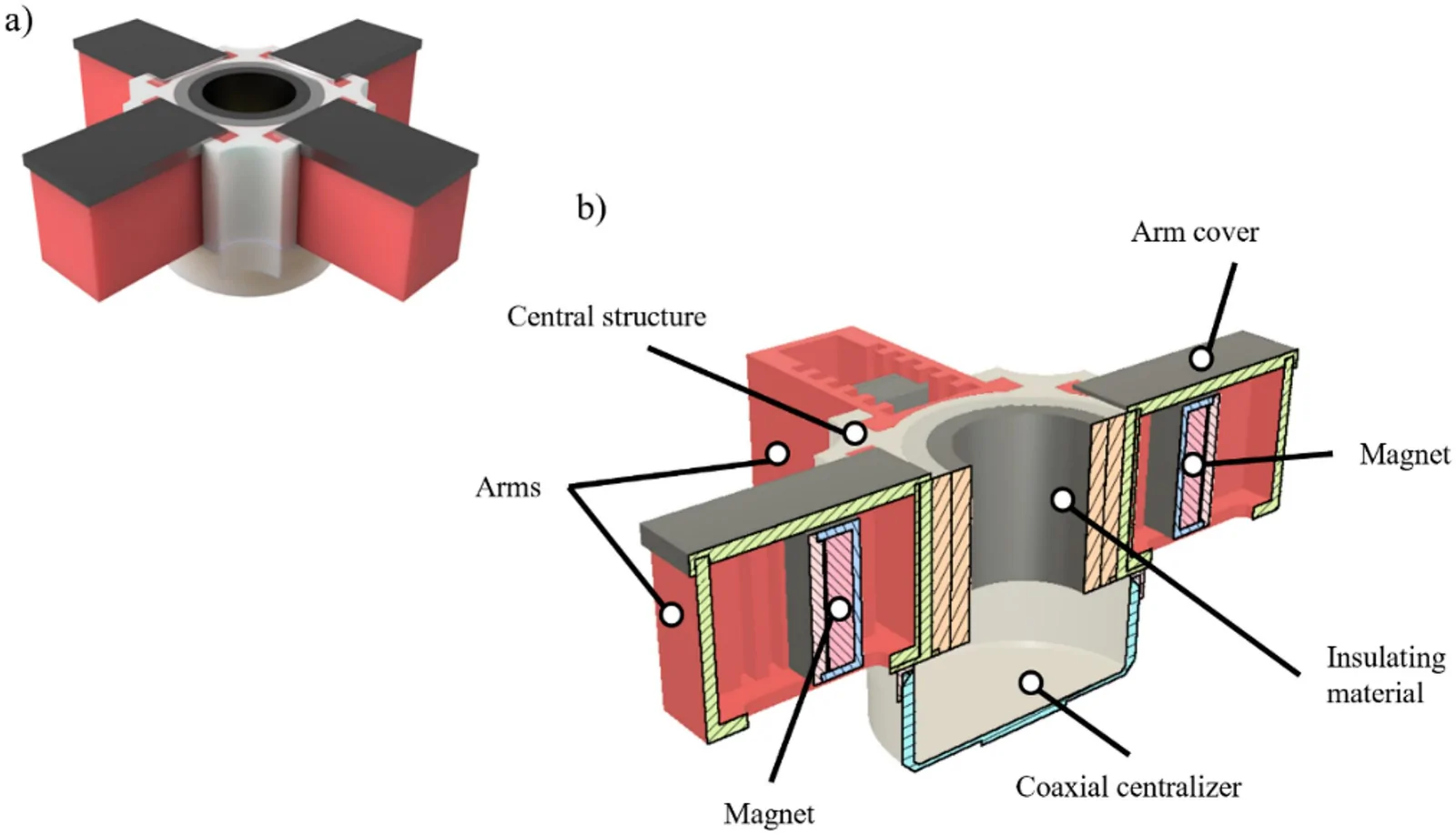
Modular accessory for applying magnetic fields in rheological tests. a) Modular system for magnetorheological characterization and b) details of the components.

*This accessory was specifically designed for the Rheotest RN 5.1 rotational rheometer, which operates with concentric cylinder geometries to evaluate rheological properties. The technical specifications reported correspond to this configuration. The dosing cup developed for this setup has an inner diameter of 37.4 mm and an outer diameter of 43 mm. The RN 5.1 system can measure viscosities ranging from 5 to 10^5^ mPa·s, with shear rates up to 2000 s^−1^.*

*Although the accessory was developed based on the Rheotest RN 5.1 configuration, it is also compatible with other column-type rotational rheometers, such as those manufactured by T.A. Instruments, PCE Instruments, and Thermo Fisher.* Fig. 2 *shows the RN 5.1 rheometer together with the designed accessory.*

**Fig. 2:**
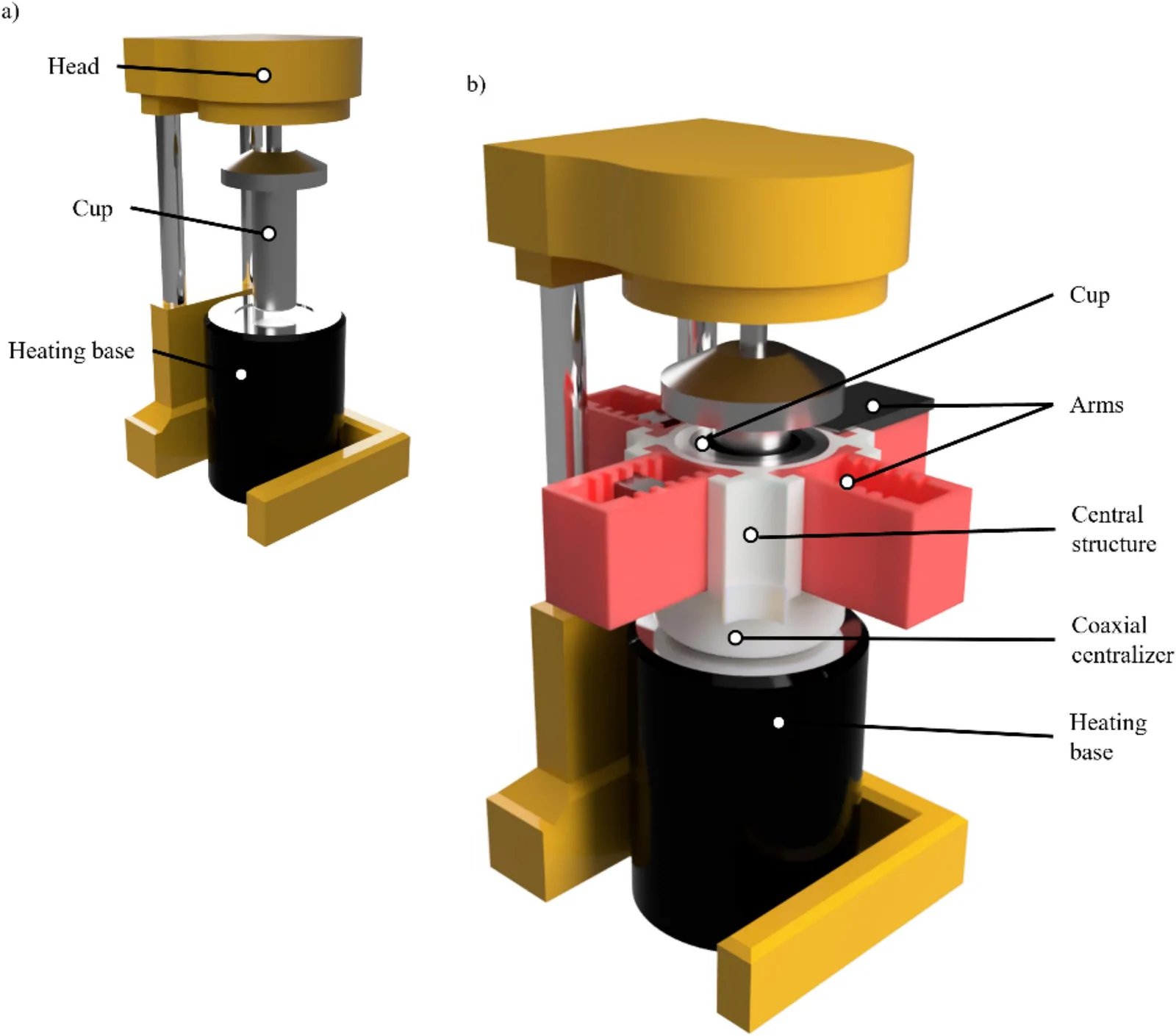
**(a**) Rheotest RN 5.1 rheometer. (b) Schematic of the accessory position in the reference rheometer.

*An additional assembly was developed to characterize the magnetic field generated by different magnet configurations. This gauge element positions the gaussmeter probe at predefined Cartesian coordinates and allows the vector components of the magnetic field to be determined in the region corresponding to the fluid volume.*

*The magnet-holding capsules were designed with dimensions of 46 × 21 × 10 mm, matching the size of the neodymium magnets used in the setup. This configuration ensures full coverage of the deformation region defined by the selected rheometric geometry. The capsule design can be easily adapted to accommodate other commercial magnet sizes, making the system compatible with different rotational rheometers or viscometers.*

*The gauge element consists of a central structure to which two arms can be attached at the same relative distance used in the rheometer accessory. Its top and lateral inlets guide the probe to selected measurement points, enabling the magnetic field distribution in the region of interest to be evaluated before rheological testing.* Fig. 3 *shows the gauge element, its components, and the locations of the arms and magnets.*

**Fig. 3:**
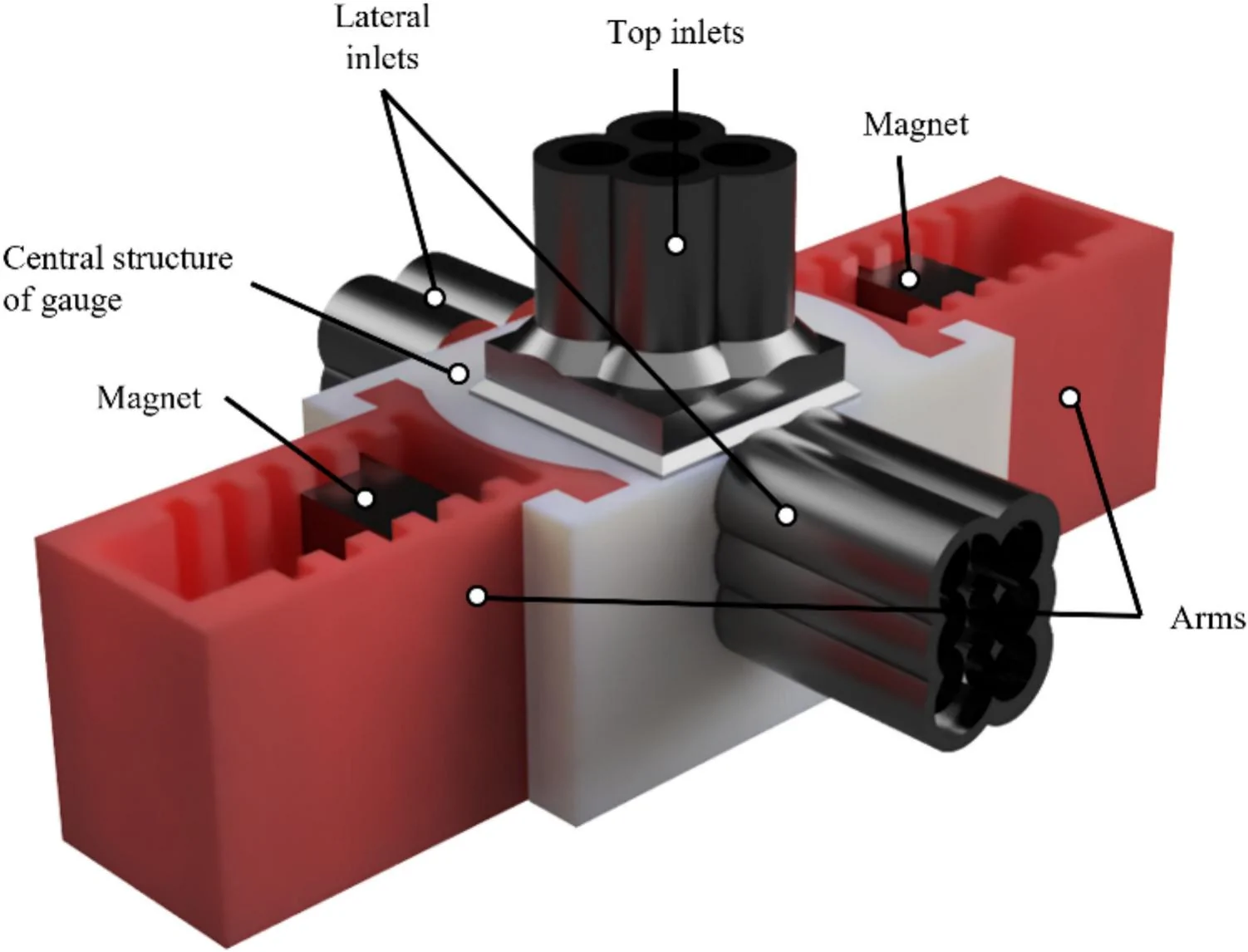
Gauge element coupled to arms and magnets.

*Additionally, protective covers were designed to be mounted on the arms of the accessory to prevent accidental displacement of the magnets caused by strong repulsive or attractive magnetic forces from nearby metallic objects.*

*An additional assembly was developed to complement the magnetic field characterization under different magnet configurations. Using this assembly, the measuring probe connected to a gaussmeter could be precisely positioned for a maximum of two arms simultaneously. Using this system, i.e., the “gauge element,” the vector configuration of the magnetic field could be determined for each experimental arrangement of the magnets. The measurements were then added in a three-dimensional space defined by the Cartesian coordinates x, y, and z.*

*The gauging element has a central structure, to which two arms can be affixed at a distance equivalent to that of the accessory for applying magnetic fields in the rheometer. This configuration replicates the positioning conditions of the magnets and is equipped with top and lateral inlets. These inlets facilitate the precise placement of the measuring probe at designated coordinates within the space containing the ferrofluid. Additionally, the magnetic field within the region of interest can be accurately evaluated using this configuration.* Fig. 3 *shows the gauge element, its components, and the locations of arms and magnets.*

*Using the side inlets, measurements can be performed in the xy plane at 12 positions, corresponding to the top of a single quadrant of the gauge. Owing to the assembly symmetry, up to 40 measurements can be performed for the entire area containing the ferrofluid. Three variants of top inlets were also designed to measure the xz and yz planes at different depths in the z axis. These depths coincide with the positions of lateral inlets and facilitate the characterization of applied magnetic fields in the three planes of the coordinate system.* Fig. 4 *shows the magnetic-field measurement points accessed by the gauge element.*

**Fig. 4:**
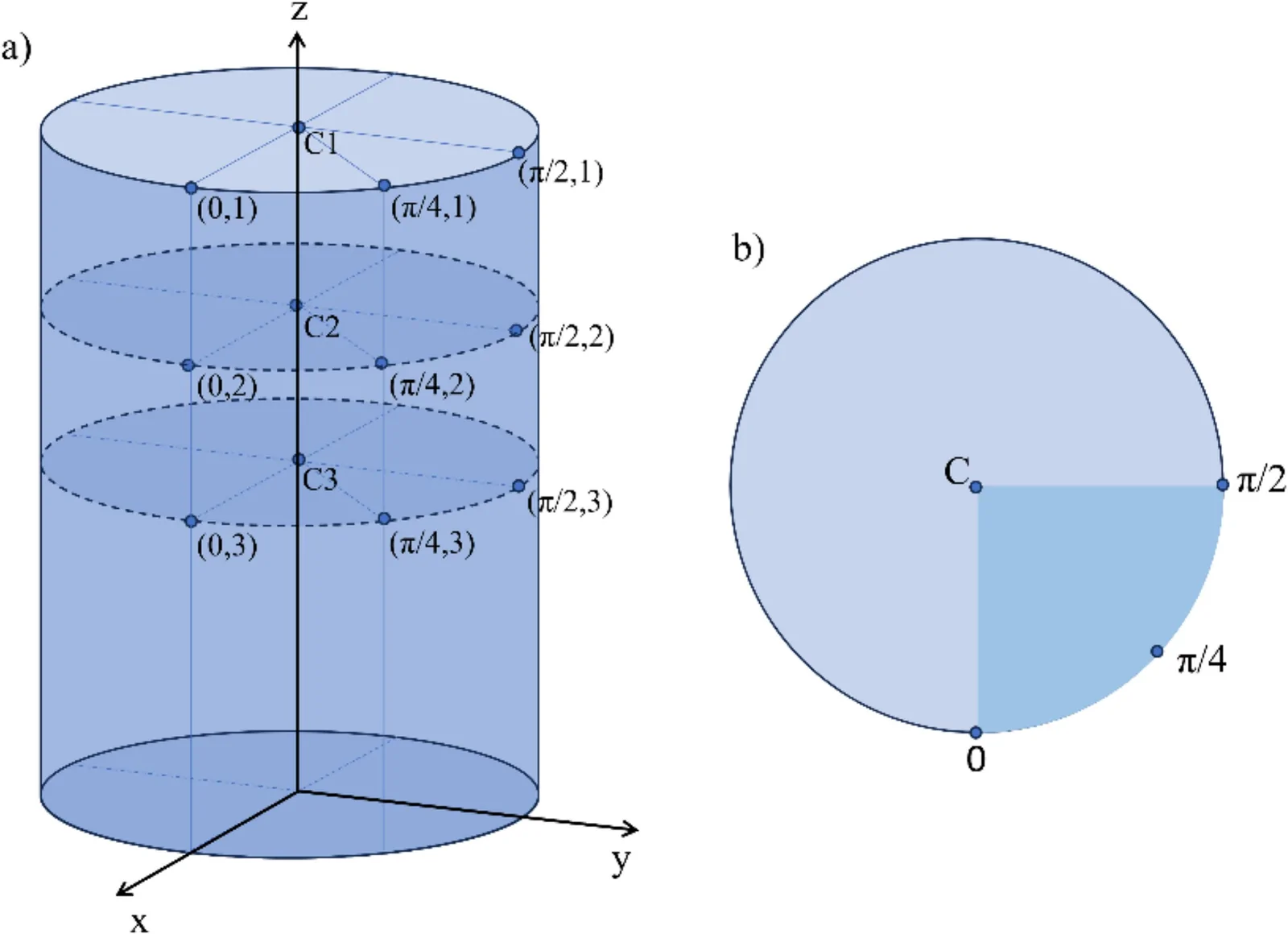
Magnetic field measurement points. a) Spatial position of the field measurement points and b) measurement points in top view.

*The gauge element is used independently to characterize the magnetic field before testing and is not mounted on the rheometer during rheological measurements. During testing, only the magnetic-field application accessory is positioned around the rheometer cup. The gauge reproduces the relative magnet positions of the accessory and provides defined probe locations for measuring the magnetic field components in the region corresponding to the fluid volume.*

*The central structure of the gauge, arms, fixed side inlets, and interchangeable top inlets enabled the precise positioning of magnets, ensuring consistent and reproducible magnetic field measurements at the specified coordinates.*

*The gauge component is used independently to characterize the magnetic field generated by each magnet configuration before testing. During rheological measurements, only the magnetic-field application accessory is positioned around the rheometer cup. The gauge element replicates the relative position of the magnets in the accessory and provides defined probe locations for measuring the magnetic field components in the region corresponding to the fluid volume.*

## 3 Design files summary

### 3.1 Bill of materials summary

*The device was fabricated by fused filament fabrication, an accessible and cost-effective 3D printing technique that enables rapid iteration of parts with complex geometries* [13], [14]*. Polylactic acid (PLA) was used because of its dimensional stability, biodegradability, low thermal shrinkage, and adequate mechanical strength for structural laboratory components. PLA is also compatible with conventional 3D printers and requires lower bed temperatures than many alternative materials* [15]*.*

*The components were printed in an FLSUN Super Racer 3D-FDM printer equipped with a 0.2-mm nozzle at a printing speed of 100 mm per second and a layer height of 0.2 mm. A total of 1375 g of PLA was used to form the magnetic assembly of the rheometer. The total printing time was 64 h. The remaining salient print setup parameters are listed in* Table 4*.*

**Table 4:** 3D printing parameters of the components.

Designator	#	Print layer (mm)	Walls	Filament quantity (g)	Unit print time (min)	Total print time (min)
Central structure	1	0,2	3	180	513	513
Coaxial centralizer	1	0,2	3	150	340	340
Arms	4	0,2	3	400	310	1240
Capsules	4	0,2	3	44	42	168
Capsule covers	4	0,2	3	20	4	16
Arm covers	4	0,2	3	64	47	188
Central structure of gauge	1	0,2	3	257	599	599
Axial inlet	1	0,2	3	34	98	98
Peripheral inlet	1	0,2	3	45	142	142
Top inlet 1	1	0,2	3	72	198	198
Top inlet 2	1	0,2	3	60	166	166
Top inlet 3	1	0,2	3	49	135	135

*Regarding service life, the PLA components mainly function as positioning and support elements and are not subjected to significant cyclic mechanical loading during normal room-temperature operation; therefore, fatigue is not expected to be the governing degradation mechanism. However, their durability may be affected by elevated temperature, humidity, ultraviolet radiation, chemical cleaning agents, or accidental damage. The arm slots, magnet capsules, covers, and centralizing elements should be visually inspected before use and replaced if deformation, cracking, looseness, or loss of magnet positioning accuracy is observed.*

*The assembly of the magnetic-field application accessory and the gauge element is described in the following sections.*

## 4 Assembly instructions

### 4.1 Magnetic field application accessory

***Step 1. Selection of the number of arms:*** *The number of arms depends on the geometry of the magnetic field to be generated.* Fig. 5 *illustrates the three possible configurations and the corresponding distances between magnets, from which the most suitable arrangement is selected.*

**Fig. 5:**
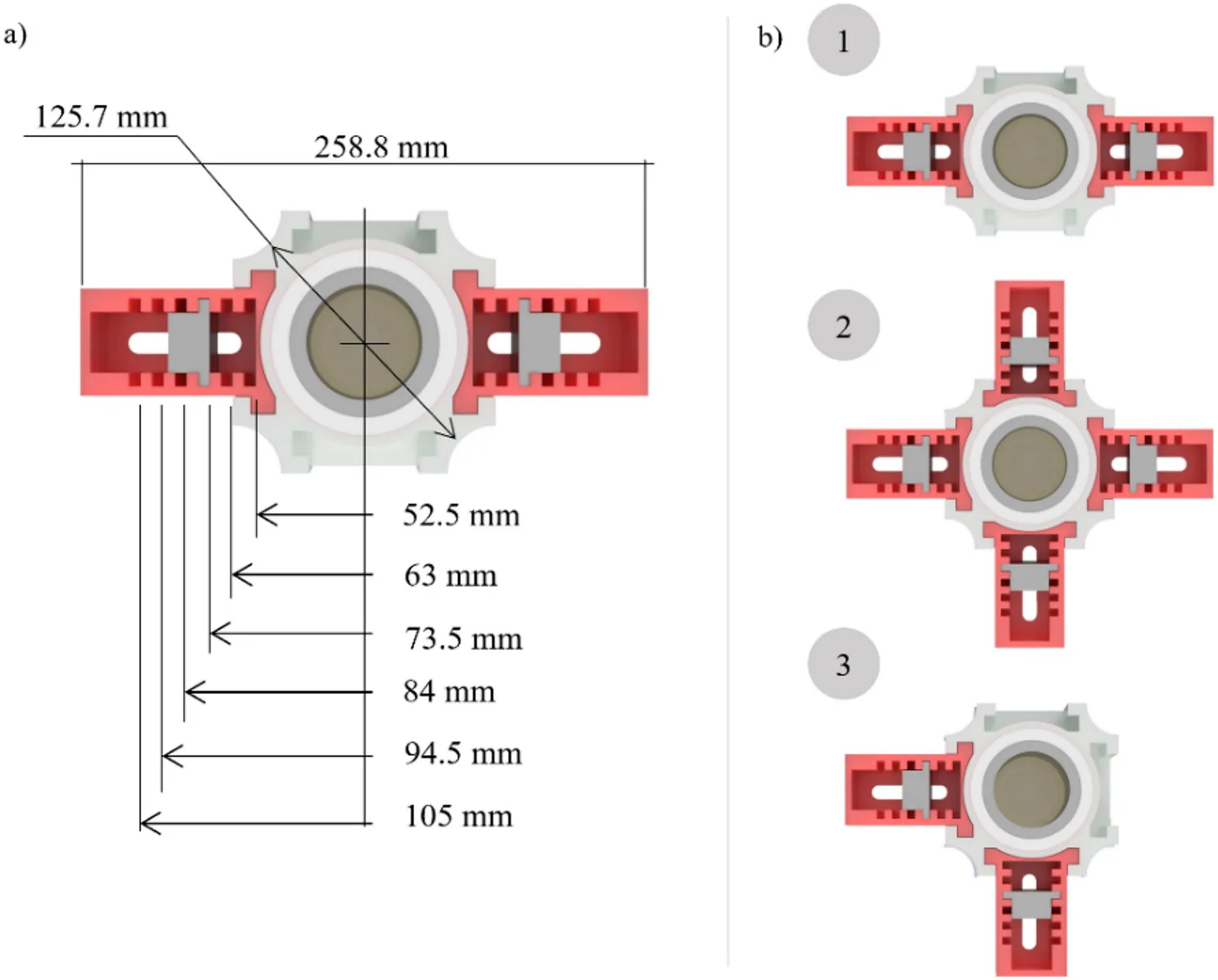
Magnet placement options. a) Distance between magnets and b) arm configuration in the assembly.

***Step 2. Magnet encapsulation:*** *Each magnet was inserted into its designated capsule and sealed—either by pressure fitting or adhesive—to prevent displacement. Prior to encapsulation, the north and south poles of each magnet were identified using the probe of a gaussmeter, ensuring their correct orientation according to the required magnetic field configuration (e.g., N–N or S–N).* Fig. 6 *shows the capsule components, their structural design, and the arrangement of the magnets.*

**Fig. 6:**
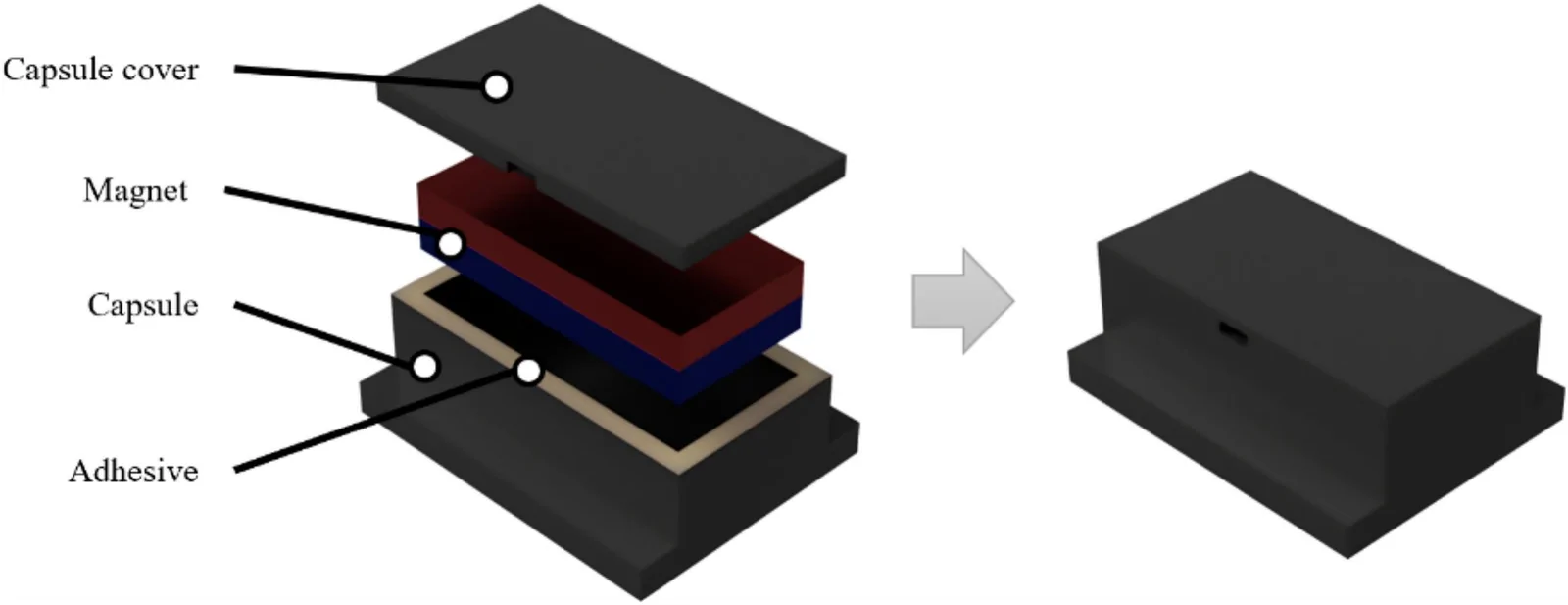
Magnet encapsulation.

***Step 3. Placement of the central structure in the centralizer:*** *The central structure was first positioned in the centralizing element to ensure proper alignment with both the rotational and symmetry axes of the fixture. As shown in* Fig. 7*, the centralizer is directly coupled to the central structure of the assembly to maintain precise concentricity during operation.*

**Fig. 7:**
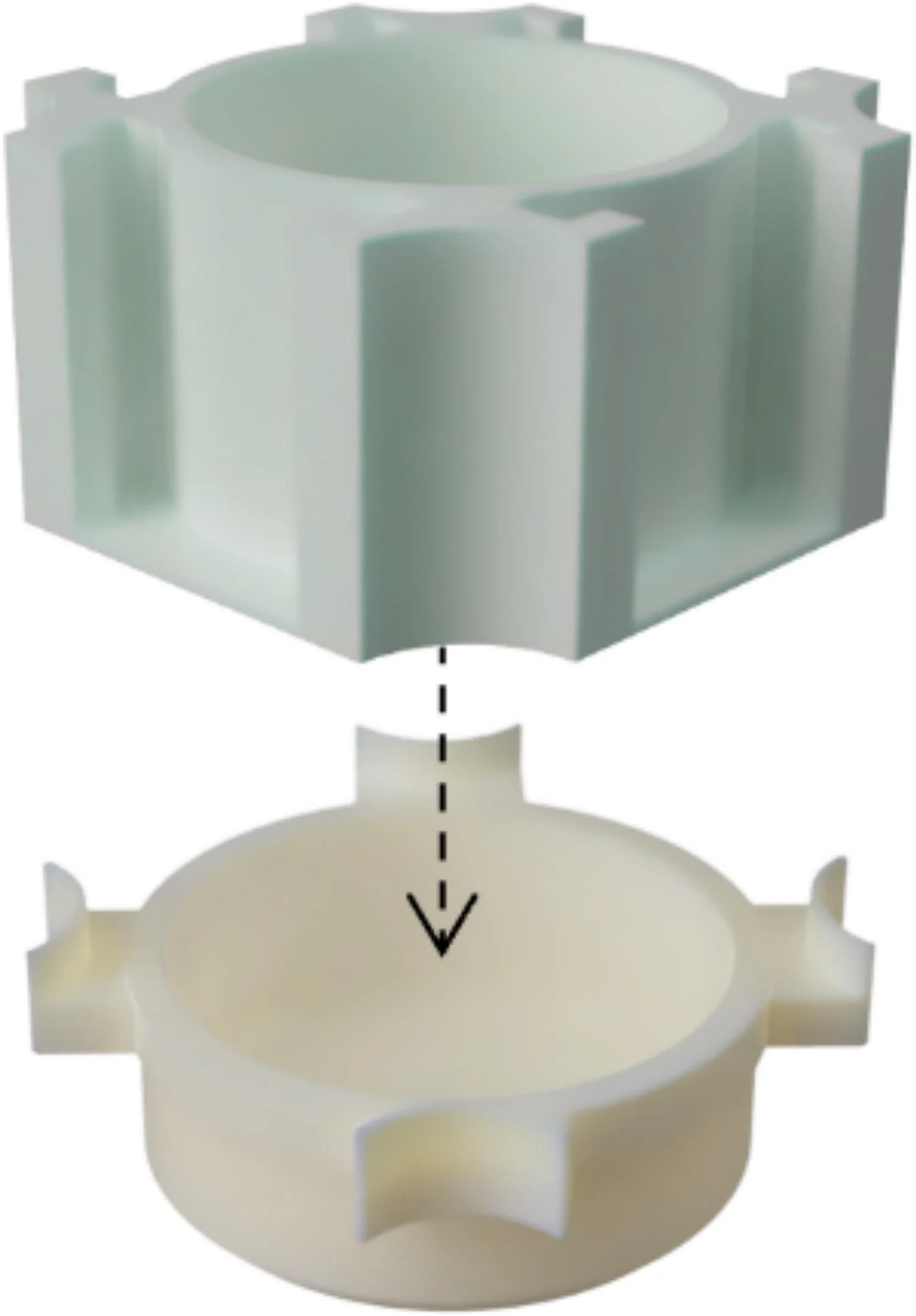
Adjustment between the centralizer and central structure.

***Step 4. Insertion of the arms into the central structure:*** *Once the central structure was aligned, the arms were inserted into their designated slots according to the predefined number and configuration.* Fig. 8 *illustrates the assembly process, highlighting the integration of the arms with the central structure.*

**Fig. 8:**
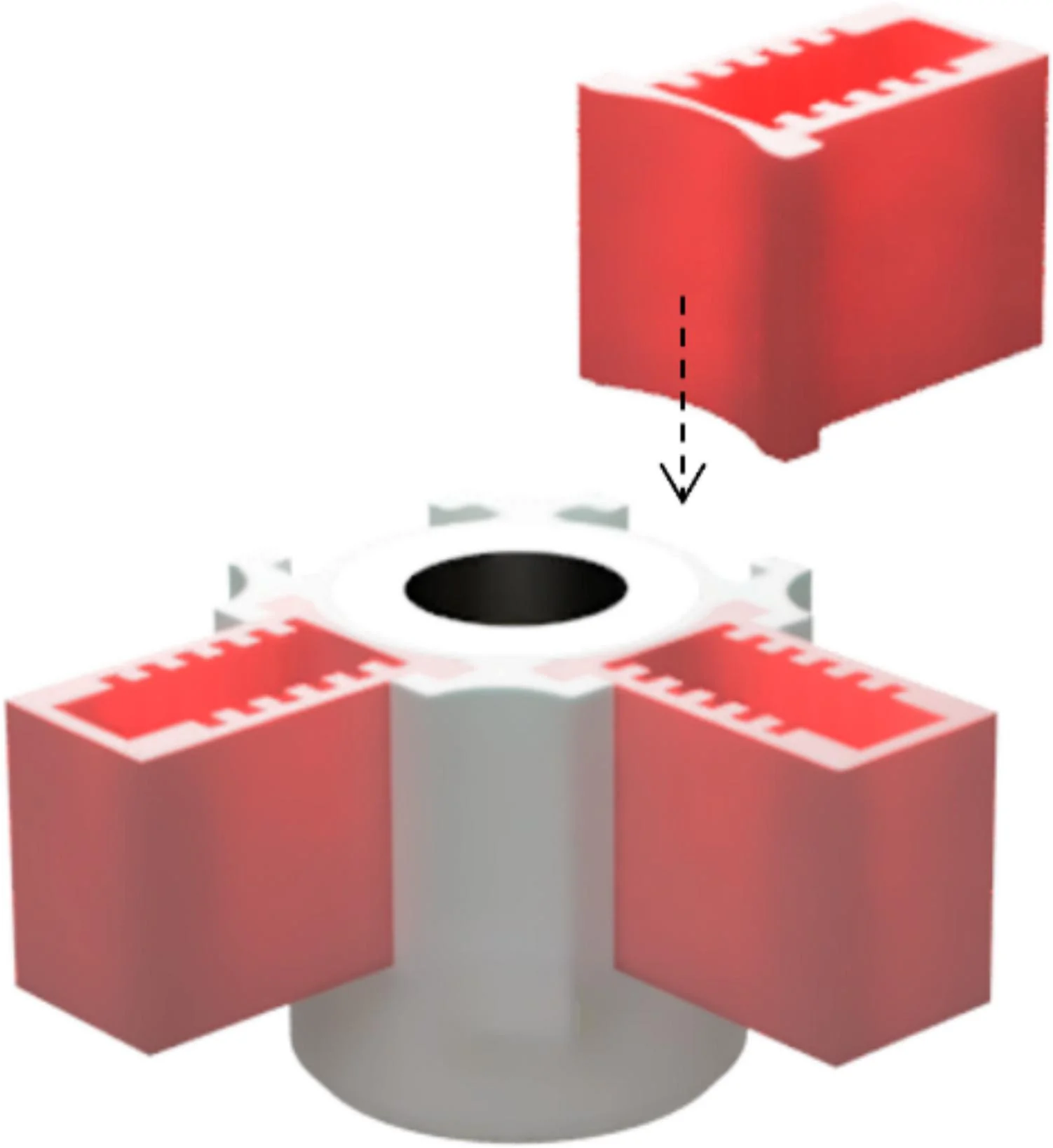
Placement of the arms on the central structure.

***Step 5. Insertion of magnets in their positions:*** *The capsules with magnets were placed in the predetermined positions along each arm. The distances between them were set using location guides based on the geometry and field intensity. This procedure is shown in* Fig. 9*.*

**Fig. 9:**
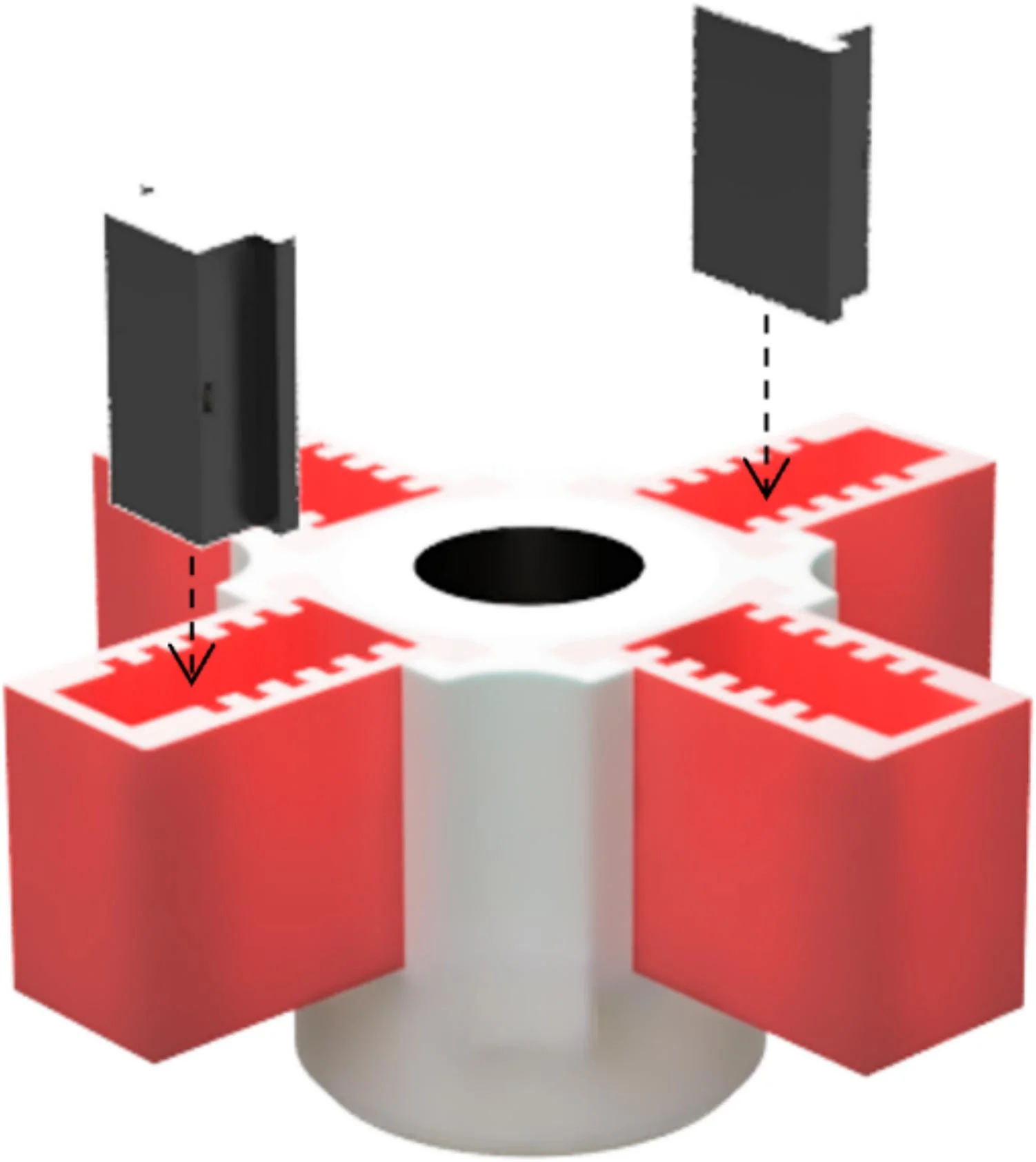
Positioning of the magnets on the arms.

***Step 6. Positioning of arm covers:*** *A cover was placed over the arms to prevent possible accidental movements of the magnets caused by the attraction or repulsion forces between them or with surrounding metallic objects.* Fig. 10 *shows the positions of covers on the arms.*

**Fig. 10:**
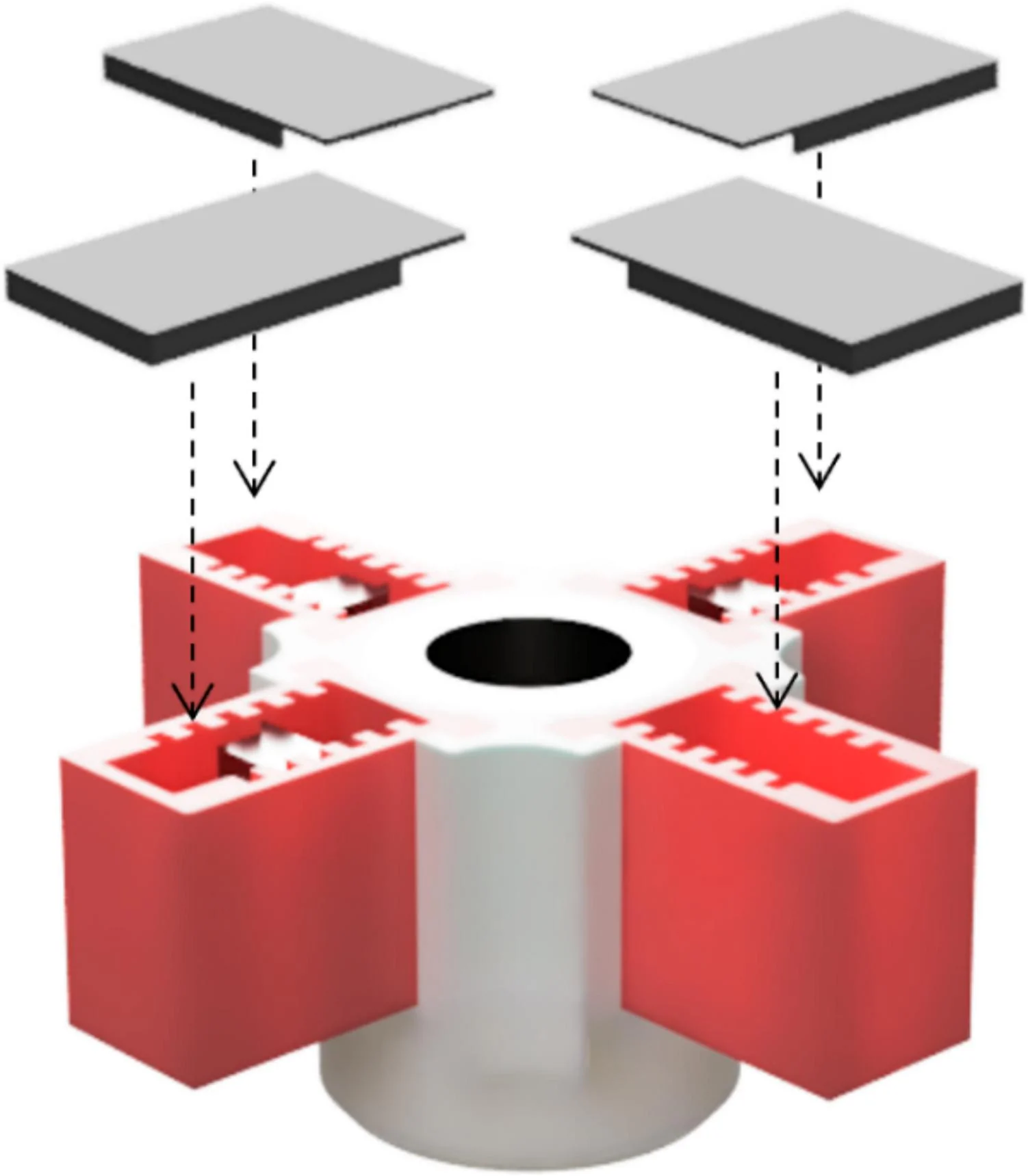
Location of cover on the arms.

### 4.2 Gauging accessory

***Step 1. Positioning of the side inlets:*** *The side inlets were fixed using an adhesive; its configuration is shown in* Fig. 11*.*

**Fig. 11:**
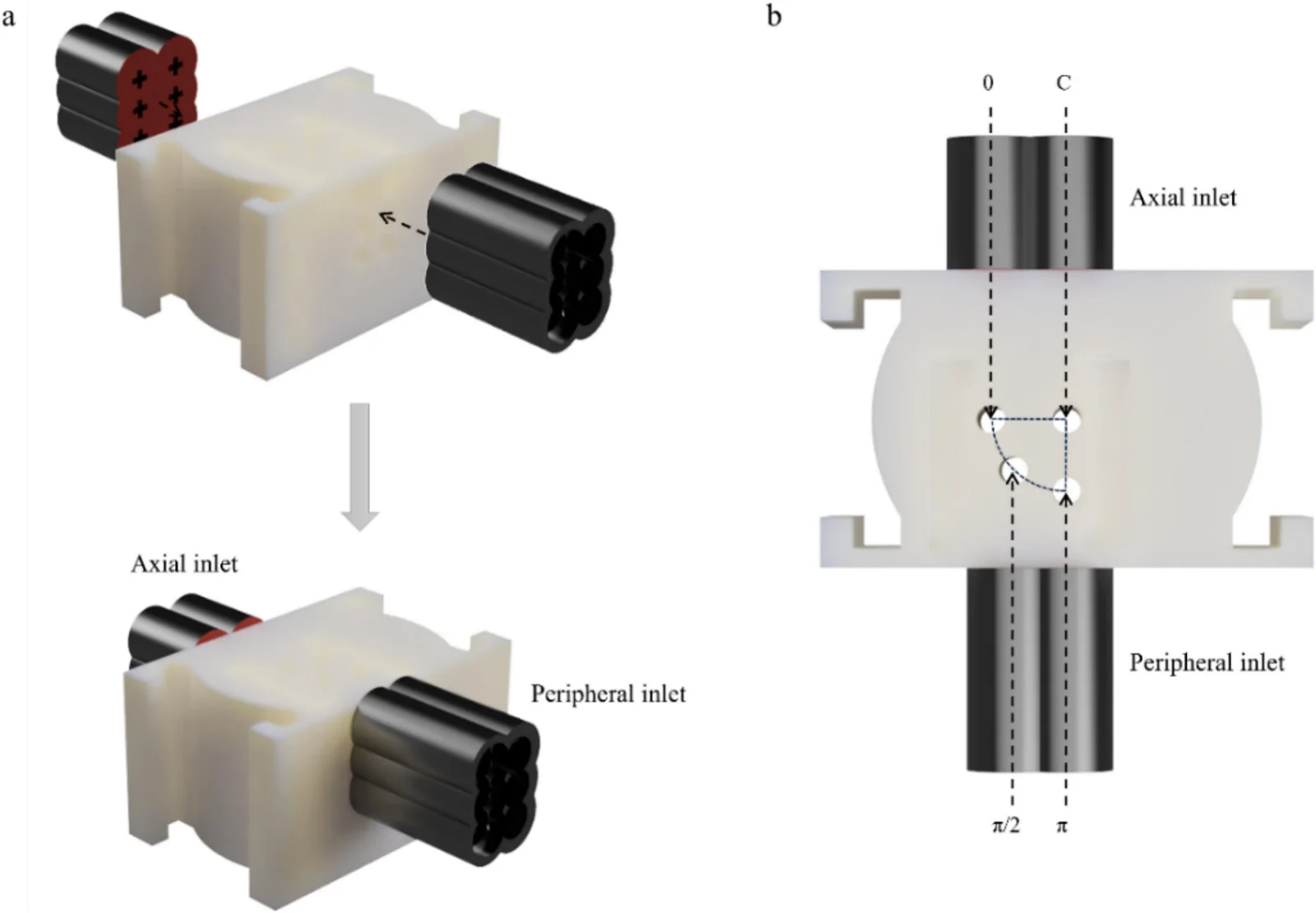
Positioning of the side inlets. a) Central structure coupled to side inlets and b) top view of inlet configuration.

***Step 2. Location of the arms:*** *The arms were inserted into the available tabs on the central structure of the gauging element, as shown in* Fig. 12*.*

**Fig. 12:**
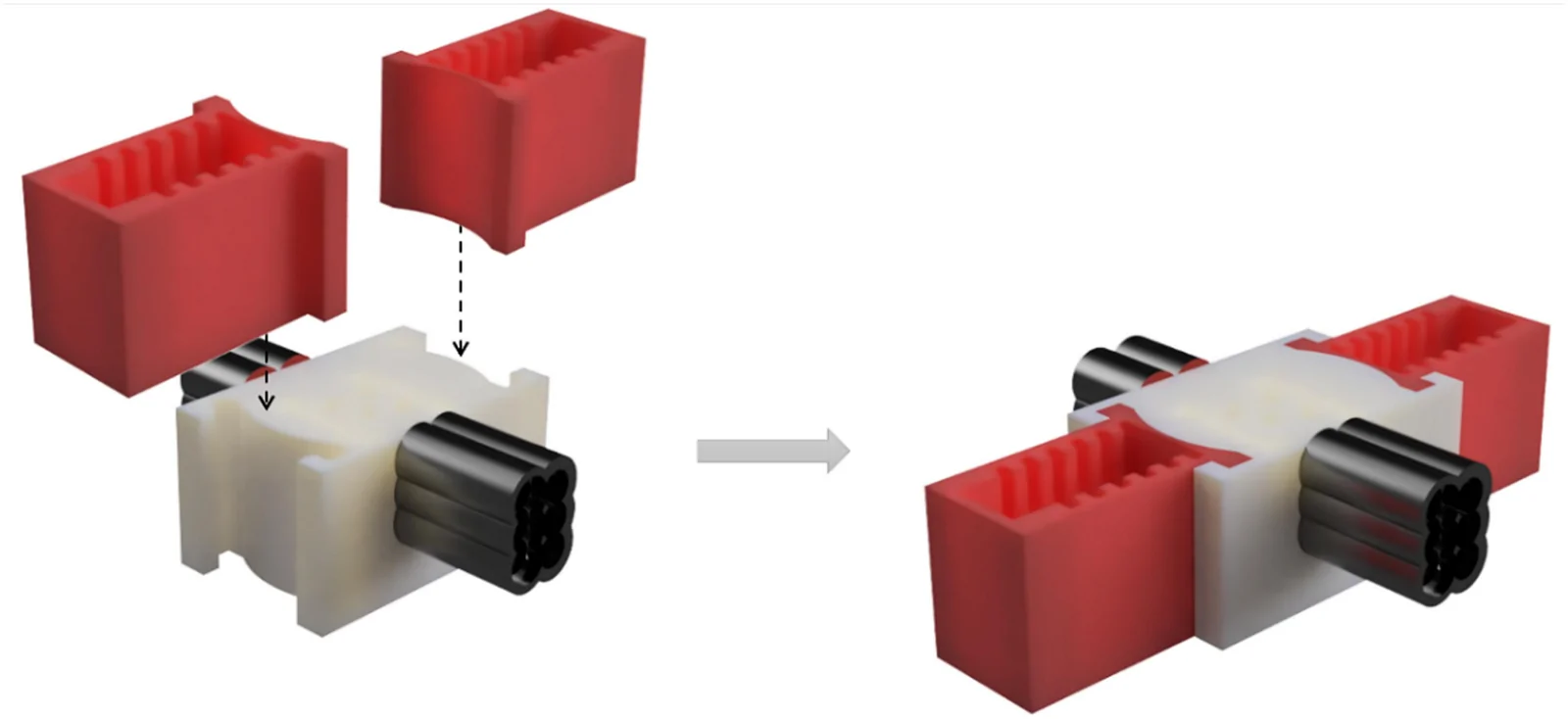
Location of the arms in the gauge assembly.

***Step 3. Top inlet location:*** *The top inlets were attached to the central structure gauge depending on the depth requirement. This ensured that the inlet channels aligned with the holes in the central gauge structure.* Fig. 13 *shows the location of the inlets on the central structure of the gauge.*

**Fig. 13:**
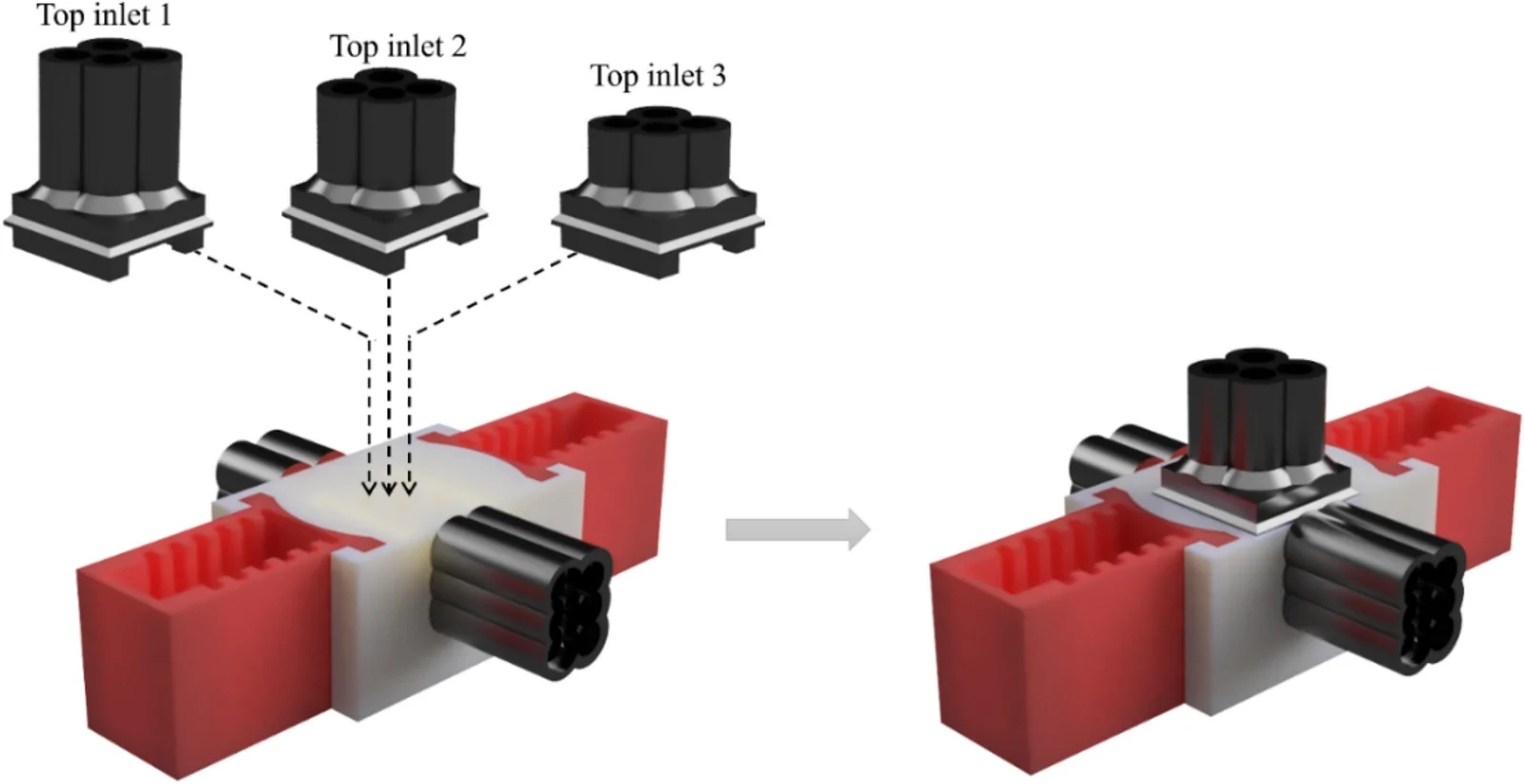
Positioning the top inlet on the gauge.

## 5 Operation instructions

### 5.1 Assembly operating instructions

***Step 1. Initial alignment check:*** *The heating base of the Rheotest rheometer was positioned according to the manufacturer’s specifications and used as a reference for alignment. The head was lowered with the dosing cup to verify that the hole in the heating base was centered relative to the cup position, ensuring preliminary alignment between the measurement geometry and the base. After verification, the head and cup were raised again to provide sufficient clearance for installing the magnetic accessories (centralizer and central structure) without interfering with the cup or the rheometer geometry.*

*It should be noted that, for other compatible rheometers, a custom centralizer would need to be designed and adapted according to the diameter of the fluid-containing cup to achieve proper coupling and alignment with the magnetic field application accessory.*

***Step 2. Dosing the test fluid:*** *The cup was then removed from the rheometer, and the volume of fluid corresponding to the selected concentric cylinder geometry was added. Then, the rotor was inserted into the rheometer head, and the cup is repositioned with fluid.*

***Step 3: Installation of the accessory in the rheometer:*** *The protruding cylinder of the centralizer coupled with the rest of the assembly was carefully inserted into the hole of the heating base. This method ensured the precise positioning (in terms of height and centrality) of the cup, thereby ensuring its symmetrical placement within the magnetic field.*

***Step 4. Relocation of the cup:*** *The head was then lowered again with the dosing cup until it reached the bottom surface of the centralizer, which acts as a mechanical stop. In the Rheotest configuration, the depth of the centralizing element was designed to position the cup within the characterized magnetic-field region. For other compatible rheometers, this centralizing component should be redesigned or replaced by an equivalent positioning reference according to the specific cup geometry and dimensions. The complete operational procedure is shown in* Fig. 14*.*

**Fig. 14:**
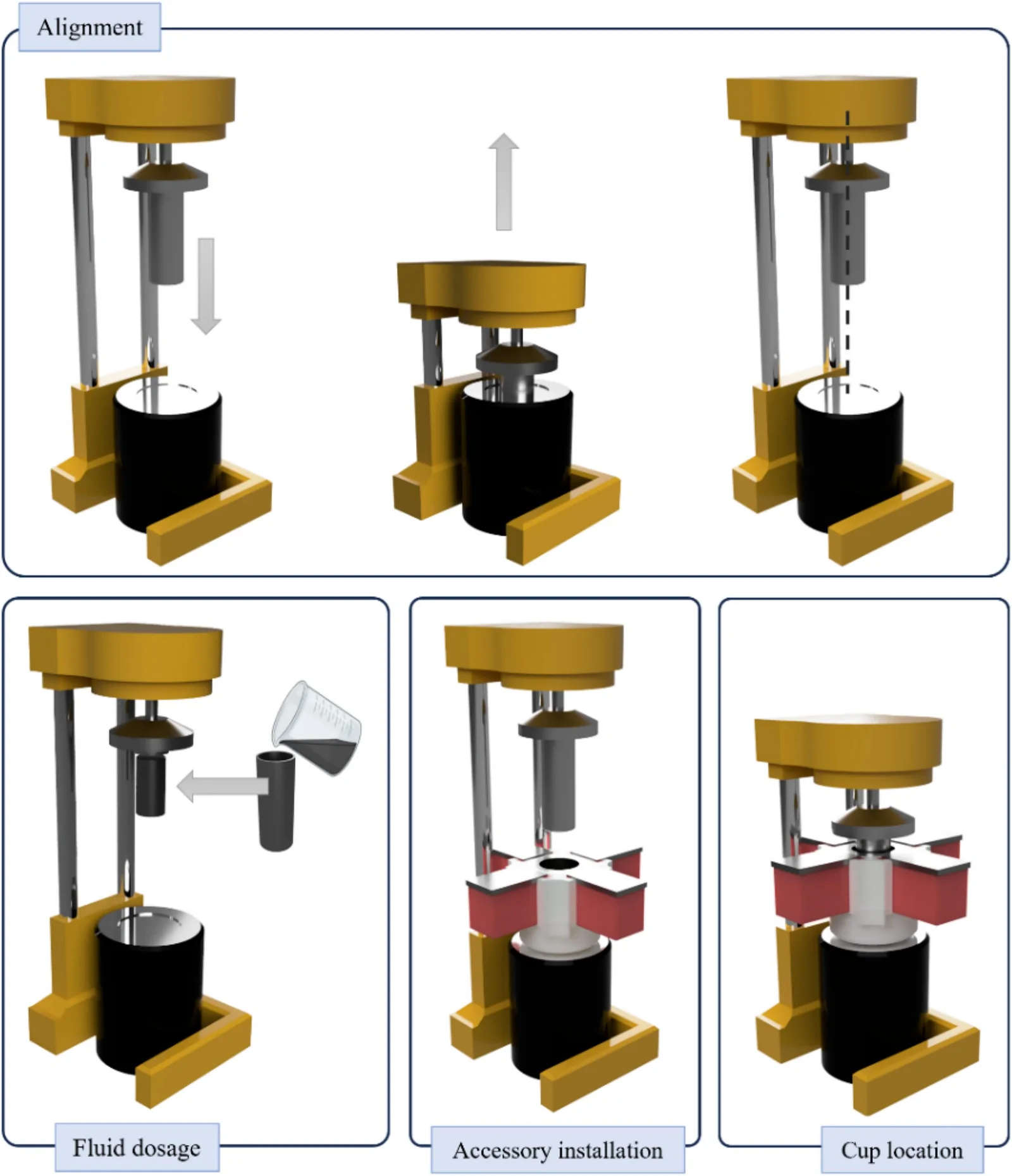
Operating procedure of the magnetic field accessory.

***Step 5. Start of the tests:*** *After appropriately configuring the system, rheological tests were conducted to characterize the ferrofluid. These tests ensured that the fluid was exposed to the desired magnetic field.*

### 5.2 Operating instructions for the gauge setup

***Step 1. Definition of measuring depth:*** *The magnetic field intensity was measured at controlled depths selected according to the experimental requirements and the twelve available probe positions defined for the gaussmeter sensor. To perform measurements at different elevations within the fluid volume, three interchangeable top inlets were used, each designed for a specific measuring depth. The spatial location of the measuring probe within the fluid domain is illustrated in* Fig. 4*.*

***Step 2. Location of the probe:*** *The gaussmeter probe was located in the corresponding slots of the inlets, allowing perpendicular measurements on the xy, xz, and yz planes.* Fig. 15 *shows the configuration of the measurement points and corresponding inlets for each plane.*

**Fig. 15:**
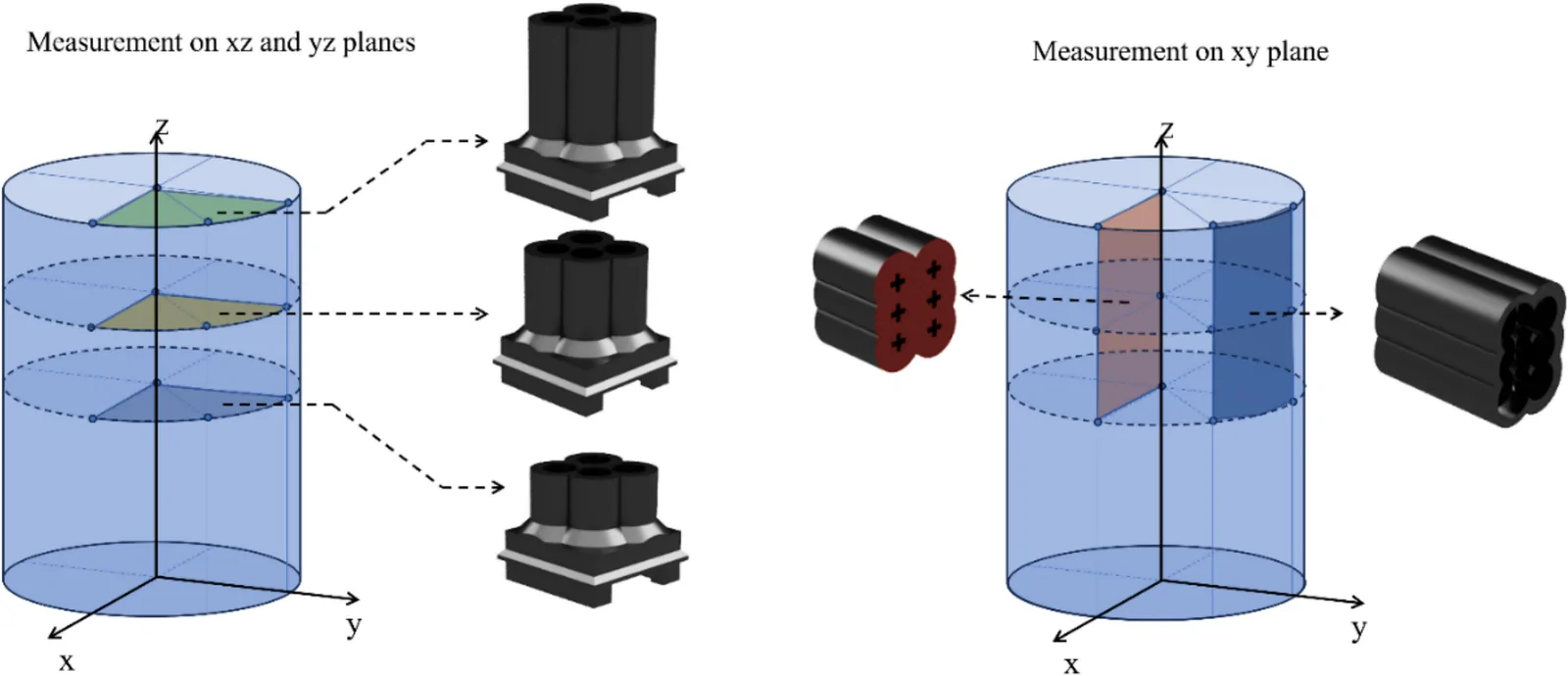
Inlets associated with different measurement points in the xy, xz, and yz planes. a) Measurement on the xz and yz planes and b) on the xy plane.

Fig. 16 *shows the operating procedure of the gauging system in accordance with the previously outlined steps.*

**Fig. 16:**
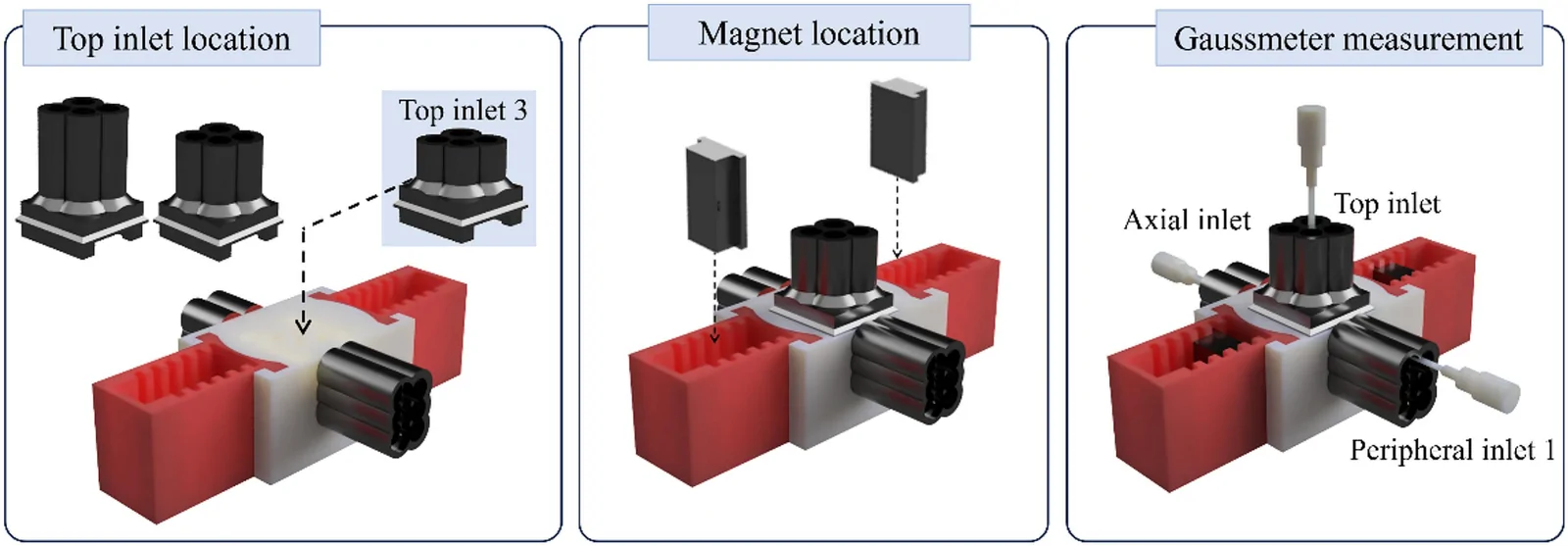
Procedure for measuring the applied magnetic field.

***Step 3. Calculation of the resulting magnetic field intensity:*** *The magnitude of the resulting magnetic field was calculated by the vector combination of the components in the three planes using Eq.* (1)*:*

(1) $$\left\|H\right\|=\sqrt{{\left(H_{\mathrm{xy}}\right)}^{2}+{\left(H_{\mathrm{xz}}\right)}^{2}+{\left(H_{\mathrm{yz}}\right)}^{2}}$$

*where* ‖H‖ *is the magnitude of the applied field and H_xy_, H_xz_ and H_yz_ are the measured intensities in the corresponding planes.*

## 6 Validation and characterization

*To validate the assembly functionality, rheological tests were performed using magnetite ferrofluids in an ethylene glycol base (Fe_3_O_4_/EG) at six volumetric concentrations: 0.01, 0.05, 0.1, 0.3, 0.5, and 1 % Cv. This range was selected because it is representative of magnetic nanofluid formulations for thermal-fluid applications, where viscosity affects handling, pumping, convective transport, and heat-transfer performance. Accordingly, the objective was not to evaluate viscoelasticity-dominated behavior, but to determine whether the assembly could detect field-induced changes in the flow response and Bingham-type parameters within an application-relevant concentration range. The ferrofluids contained 60*–*100 nm particles and are referred to here as magnetic nanofluids or ferrofluids because the dispersed magnetic phase remains within the nanoscale and the formulations were prepared as stable liquid suspensions.*

*The validation was designed as a functional evaluation of the accessory, not as a full metrological comparison with a commercial magnetorheological system. The accessory does not modify the rheometer sensing principle or torque measurement system; it provides a controlled magnetic-field configuration around the measuring geometry. Thus, measurement accuracy remains governed by the commercial rheometer calibration and specifications, while this study evaluates whether the accessory can generate characterized magnetic-field conditions and detect reproducible field-induced changes in magnetic nanofluids.*

*The shear stress versus shear rate profiles were recorded using Assembly 1, corresponding to the configuration illustrated in* Fig. 5*(b). A preconditioning phase was applied at a constant shear rate of 20 s^−1^ for 10 s, followed by a ramp-up from 0 to 200 s^−1^ in 60 s, and a subsequent ramp-down from 200 to 0 s^−1^ in 60 s. To ensure the reliability of the results and allow for statistical evaluation, each test was replicated three times.* Fig. 17 *shows the rheological characterization process.*

**Fig. 17:**
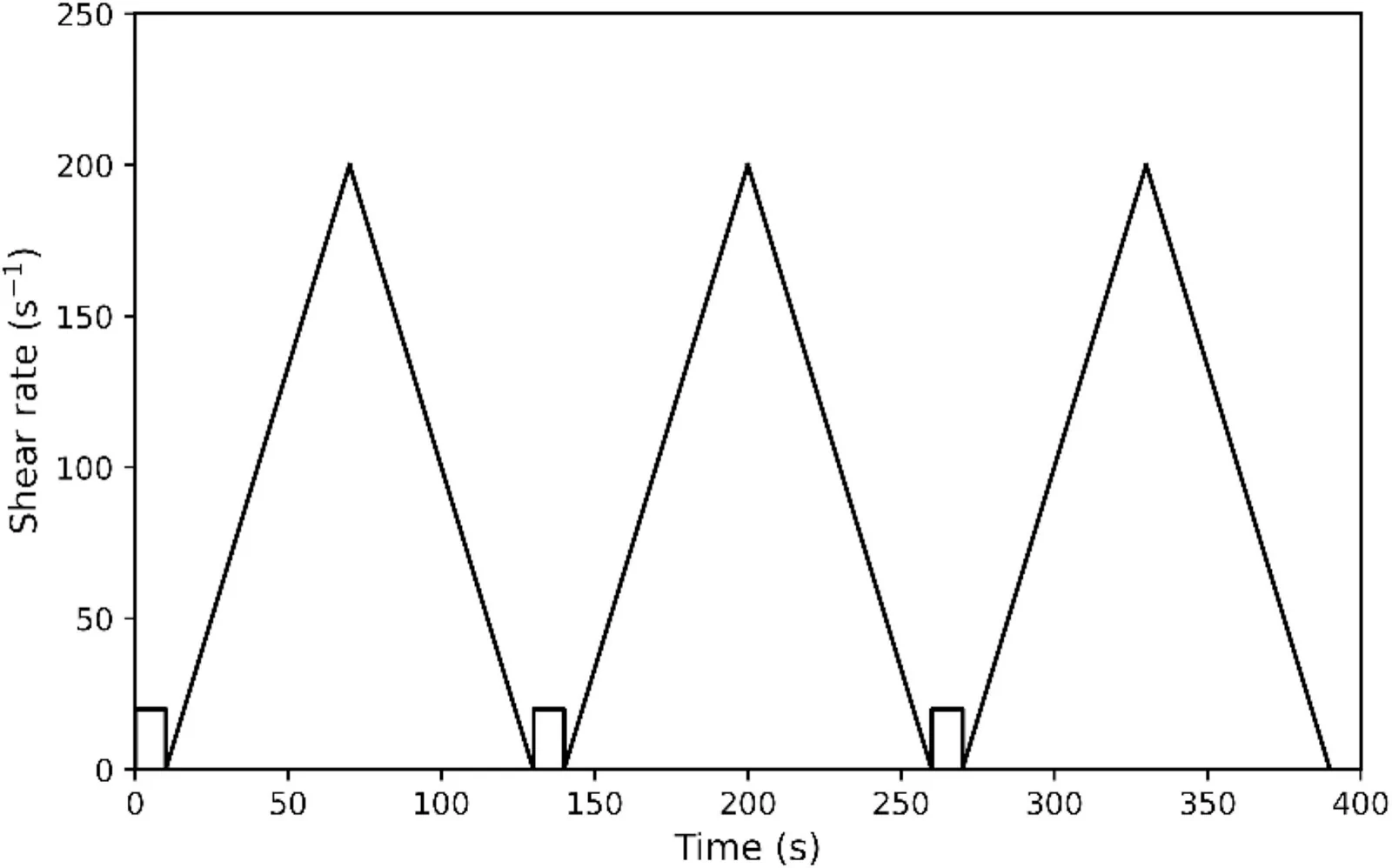
Shear rate curves applied vs time.

*The magnetic field intensities generated by the magnets positioned at different distances (as shown in* Fig. 5*) were calculated using the magnetic gauge system, and the corresponding values are summarized in* Table 5*.*

**Table 5:** Measured applied magnetic field intensities for different magnet configurations (G).

	C				0				π/2				π/4			
	$H_{\mathrm{xz}}$	$H_{\mathrm{yz}}$	$H_{\mathrm{xy}}$	‖H‖	$H_{\mathrm{xz}}$	$H_{\mathrm{yz}}$	$H_{\mathrm{xy}}$	‖H‖	$H_{\mathrm{xz}}$	$H_{\mathrm{yz}}$	$H_{\mathrm{xy}}$	‖H‖	$H_{\mathrm{xz}}$	$H_{\mathrm{yz}}$	$H_{\mathrm{xy}}$	‖H‖
1	27,3	3,4	4,4	27,9	31,7	3,6	2,1	32,0	24,5	2,5	0,0	24,6	27,7	6,7	5,4	29,0
2	36,5	4,9	6,4	37,4	43,3	4,7	4,4	43,8	32,1	3,5	0,1	32,3	37,5	10,2	6,5	39,4
3	49,7	6,1	8,4	50,8	62,1	6,4	7,6	62,9	43,2	4,8	0,3	43,5	51,4	14,5	9,9	54,3
4	70,3	9,0	12,4	72,0	93,1	9,2	15,2	94,8	59,5	6,2	0,6	59,8	74,3	23,4	15,5	79,4
5	103,4	12,6	18,2	105,7	147,9	14,3	34,3	152,5	84,5	9,6	1,1	85,1	110,8	39,9	25,4	120,5
6	163,3	20,0	29,0	167,1	262,3	22,8	58,1	269,6	122,7	13,1	2,8	123,4	168,4	74,9	43,6	189,4

*The applied magnetic field was not assumed to be spatially uniform. Instead, its distribution was characterized with the gauge element by positioning the gaussmeter probe at predefined coordinates in the region corresponding to the fluid volume. For each magnet configuration, the measured components were combined vectorially to estimate the resulting field magnitude. The values in* Table 5 *correspond to the characterized field intensities used as reference conditions for the rheological tests.*

*The magnetic field intensities reported in* Table 5 *were measured before rheological testing for each magnet configuration. Temporal variations during each run were not directly monitored; however, the tests were performed at room temperature, without active heating, and using short measurement sequences, so significant thermally induced field variations were not expected. The magnet capsules and arm covers mechanically constrained the magnets, reducing possible displacement caused by vibration or magnetic attraction/repulsion during spindle rotation. Continuous field monitoring and quantitative temporal stability assessment remain limitations to be addressed in future work.*

Fig. 18 *shows the average shear stress versus shear rate curves obtained for each ferrofluid concentration under different applied magnetic field intensities. Each curve represents the mean response calculated from three experimental replicates for a specific solid concentration and magnetic field condition.*

**Fig. 18:**
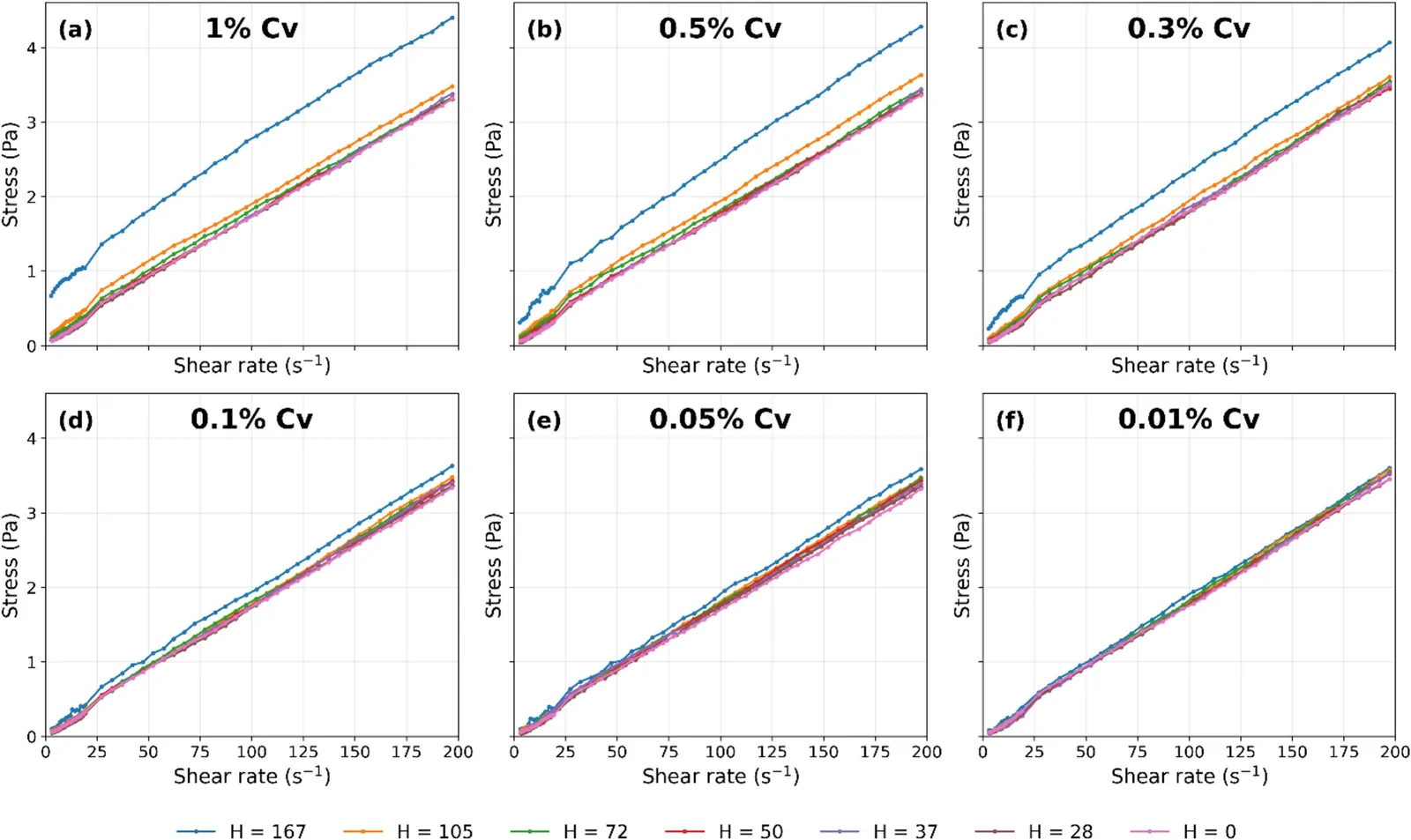
Shear stress vs. shear rate curves for different applied magnetic field intensities.

*The average shear stress versus shear rate curves showed a Bingham plastic response, as described by Equation* (2)*. This behavior is supported by the approximately linear relationship between shear stress and shear rate after flow onset and by the non-zero stress intercept associated with the apparent yield stress, τ_0_. At the lowest concentration, 0.01 % Cv, the curves were not clearly differentiated by the applied magnetic field, indicating minor field-induced changes under highly diluted conditions. As concentration increased, the curves shifted toward higher stress values mainly through an increase in τ_0_, while their slopes remained relatively similar. This indicates that the applied magnetic field mainly affected the stress offset of the Bingham-type response rather than producing large changes in plastic viscosity.*

(2) $$\tau =\mu \dot{\gamma }+\tau_{o},$$

*Here, τ is the shear stress (Pa), μ is the plastic viscosity (Pa·s), γ̇ is the shear rate (s − 1), and τ_0_ is the apparent yield stress (Pa).*

*Plastic viscosity and apparent yield stress were obtained by fitting the shear stress versus shear rate data to the Bingham model and were analyzed as functions of applied magnetic field intensity for each ferrofluid concentration (* Fig. 19 *). The plotted values are means from three experimental replicates, error bars represent the standard deviation, and linear regressions are included as descriptive trends. Overall, plastic viscosity remained close to 17 mPa·s, indicating only small variations with field intensity. In contrast, τ_0_ increased more noticeably with the applied field, particularly at higher solid concentrations and stronger magnetic fields, indicating that the field mainly modified the stress offset while the plastic viscosity remained approximately constant.*

**Fig. 19:**
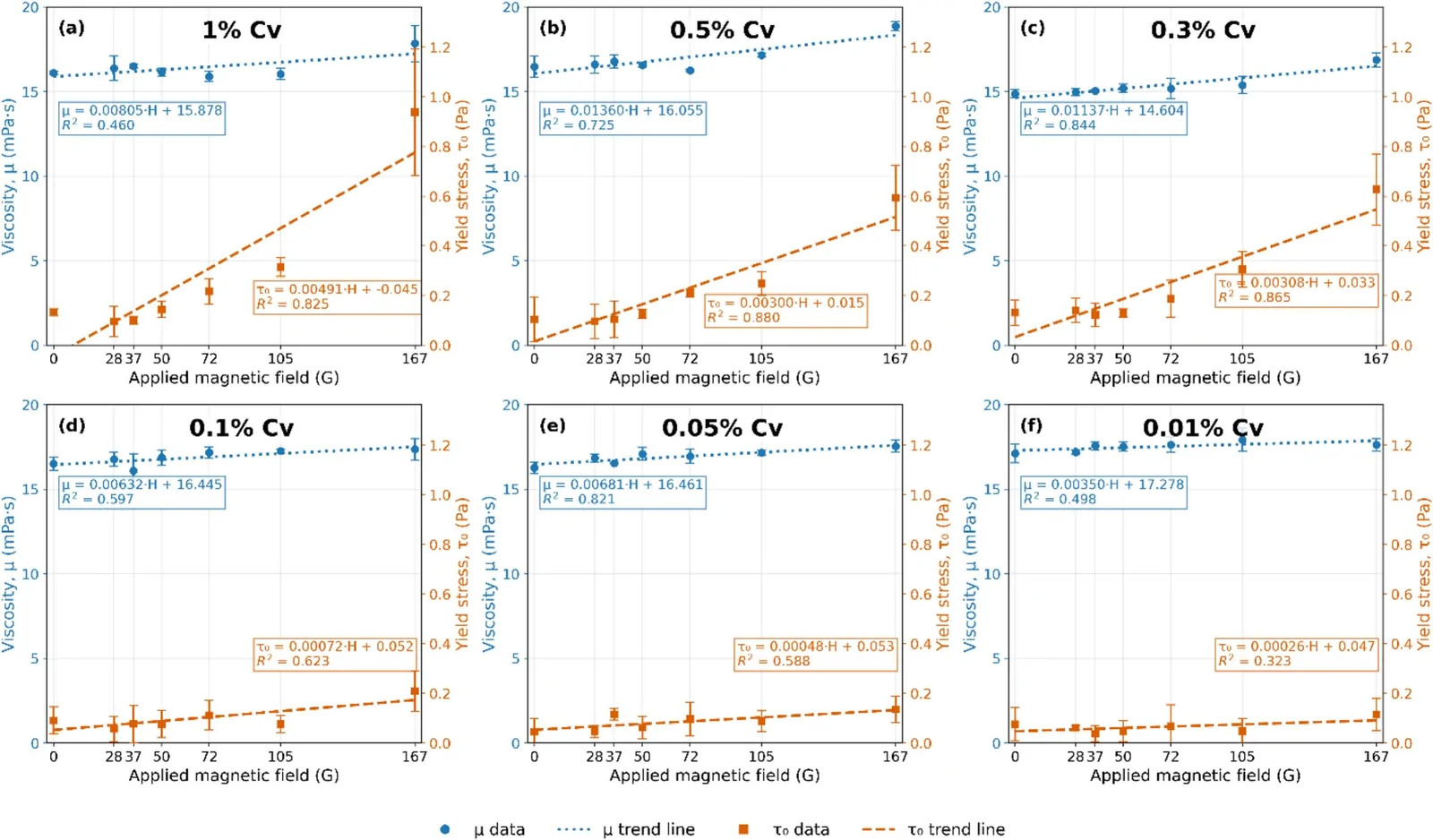
Dependence of plastic viscosity and apparent yield stress on the applied magnetic field intensity.

*Plastic viscosity and apparent yield stress were also evaluated as functions of solid concentration at each fixed magnetic field intensity (*Fig. 20*). For most field conditions, plastic viscosity remained within a narrow range of approximately 16*–*18 mPa·s, indicating that concentration did not substantially modify the viscous contribution within the evaluated range. Thus, the base fluid and overall suspension viscosity mainly determined the slope of the Bingham-type curves.*

**Fig. 20:**
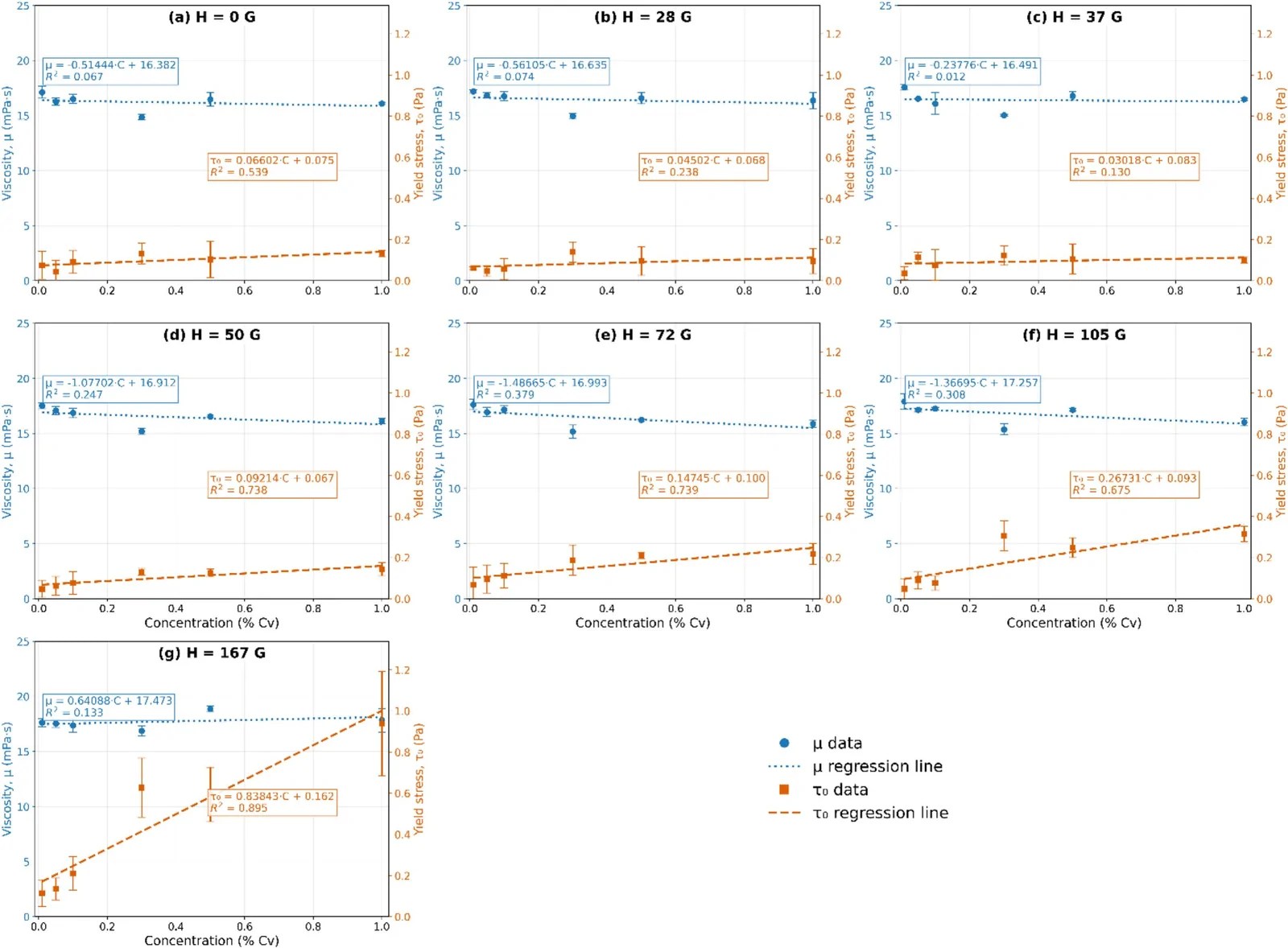
Rheological parameters as a function of ferrofluid concentration under different applied magnetic field intensities.

*In contrast, τ_0_ showed a stronger dependence on both solid concentration and magnetic field intensity. At low and intermediate fields, τ_0_ varied only slightly with concentration, suggesting weak field-induced particle structuring. At higher fields, especially 105 and 167 G, the τ_0_ regression slope increased markedly, indicating stronger particle*–*particle interactions and field-induced structures that increase the stress required to initiate flow. Therefore, the main magnetorheological effect observed in the tested ferrofluids was associated with increased apparent yield stress rather than a significant increase in plastic viscosity.*

*Overall, the results show that the developed magnetic-field application system can detect measurable magnetorheological variations in ferrofluids under characterized magnetic-field conditions. The shear stress versus shear rate curves followed a Bingham-type response, with relatively stable plastic viscosity and a τ_0_ value that was more sensitive to solid concentration and magnetic field intensity. These findings indicate that the dominant field-induced effect in the tested range was particle structuring that increased the stress required to initiate flow. Accordingly, the proposed low-cost modular assembly provides a practical platform for evaluating magnetic suspensions with different formulations, concentrations, and field configurations within the operating limits discussed above.*

*The present validation focused on room-temperature rheological measurements under different magnetic field intensities and ferrofluid concentrations. Temperature profiles of the fluid, magnets, and printed components were not measured during these tests. Therefore, although the accessory geometry allows thermal insulation or a heating jacket to be incorporated, high-temperature operation was not experimentally validated in this work and requires dedicated thermal characterization.*

## Ethics statements

*The authors confirm that they have adhered to the prevailing standards of research ethics.*

## Declaration of generative AI and AI-assisted technologies in the manuscript preparation process

*During the preparation of this work, the authors used ChatGPT (GPT-5, OpenAI) to assist in language refinement and grammatical editing. The authors reviewed and edited all generated content to ensure accuracy, scientific validity, and compliance with the journal’s ethical standards. The authors take full responsibility for the integrity and originality of the final manuscript.*

## CRediT authorship contribution statement

**Juan José Alcalde-Castro:** Writing – review & editing, Writing – original draft, Visualization, Methodology, Investigation. **Wilmer Saldarriaga-Agudelo:** Writing – review & editing, Supervision, Methodology.

## Declaration of competing interest

The authors declare that they have no known competing financial interests or personal relationships that could have appeared to influence the work reported in this paper.

## References

[1] Kole M., Khandekar S. (2021). Engineering applications of ferrofluids: A review. J. Magn. Magn. Mater..

[2] Ossege F.E.L., Gontijo R.G., de Paula A.S. (2024). Dynamical analysis of a ferrofluid subjected to oscillatory field and shear rates: Applications to magnetic hyperthermia. J. Magn. Magn. Mater..

[3] Sahoo R., Ussa-Aldana P., Lancon D., Rondelez F., Morillas J.R., Hidalgo-Alvarez R., de Vicente J. (2022). Design of smart lubricants using the inverse ferrofluid approach. Tribol. Int..

[4] Sarvar S., Rashidi S., Rafee R. (2023). A brief review of the application of ferrofluids and magnetic fields in solar energy systems. J. Magn. Magn. Mater..

[5] Abbas K., Wang X., Rasool G., Sun T., Yin G., Razzaq I. (2023). Recent developments in the application of ferrofluids with an emphasis on thermal performance and energy harvesting. J. Magn. Magn. Mater..

[6] Philip J. (2023). Magnetic nanofluids (Ferrofluids): Recent advances, applications, challenges, and future directions. Adv. Colloid Interface Sci..

[7] Dinarvand M., Abolhasani M., Hormozi F., Bahrami Z. (2023). Investigation of the effect of nanoparticle type on ferrofluid viscosity and its thermal performance in the presence and absence of a magnetic field: A new correlation. J. Magn. Magn. Mater..

[8] L. Álvarez-Gil, J. Alcalde-Castro, W. Saldarriaga-Agudelo, N. Rojas-Reyes, Low-cost modular system for magneto-rheological testing in rheometers using 3D printing , Mendeley Data, V2, 2026. 10.17632/5tpzgkvj3w.2.

[9] Rinaldi C., Chaves A., Elborai S., He X., Zahn M. (2005). Magnetic fluid rheology and flows. Curr. Opin. Colloid Interface Sci..

[10] Vinod S., Philip J. (2022). Thermal and rheological properties of magnetic nanofluids: Recent advances and future directions. Adv. Colloid Interface Sci..

[11] Joseph A., Mathew S. (2014). Ferrofluids: Synthetic strategies, stabilization, physicochemical features, characterization, and applications. ChemPlusChem.

[12] Yari B., Norouzi M., Hashemian S.M., Ghatee M., Izadifard M.H. (2025). Synthesis and characterization of a novel magnetorheological fluid based on surfactants with dual hydrophilic/hydrophobic property. Colloids Surf A Physicochem Eng Asp.

[13] Tan B., Dou J., Wen Y., Duan B., Mo H., Wei Z., Zhang J., Pan Y., Ding X., Liu N. (2024). 3D printing for explosives and propellants applications. Addit. Manuf. Front..

[14] Miller C., Patel B.A. (2024). Creative design in fused filament fabrication 3D-printed electrochemical sensors for detection of biomolecules,. Trac. Trends Anal. Chem..

[15] J. Kočí, Advanced filament guide, Prusa Research. https://blog.prusa3d.com/advanced-filament-guide_39718 (accessed October 2025).

